# A two-step, test-guided Mokken scale analysis, for nonclustered and clustered data

**DOI:** 10.1007/s11136-021-02840-2

**Published:** 2021-05-13

**Authors:** Letty Koopman, Bonne J. H. Zijlstra, L. Andries van der Ark

**Affiliations:** Research Institute of Child Development and Education, University of Amsterdam, P. O. Box 15776, 1001 NG Amsterdam, The Netherlands

**Keywords:** Automated item selection procedure, Clustered data analysis, Mokken scale analysis, Test-guided automated item selection procedure

## Abstract

**Purpose:**

Mokken scale analysis (MSA) is an attractive scaling procedure for ordinal data. MSA is frequently used in health-related quality of life research. Two of MSA's prime features are the scalability coefficients and the automated item selection procedure (AISP). The AISP partitions a (large) set of items into scales based on the observed item scores; the resulting scales can be used as measurement instruments. There exist two issues in MSA: First, point estimates, standard errors, and test statistics for scalability coefficients are inappropriate for clustered item scores, which are omnipresent in quality of life research data. Second, the AISP insufficiently takes sampling fluctuation of Mokken’s scalability coefficients into account.

**Methods:**

We solved both issues by providing point estimates and standard errors for the scalability coefficients for clustered data and by implementing a Wald-based significance test in the AISP algorithm, resulting in a test-guided AISP (T-AISP), that is available for both nonclustered and clustered test scores.

**Results:**

We integrated the T-AISP into a two-step, test-guided MSA for scale construction, to guide the analysis for nonclustered and clustered data. The first step is performing a T-AISP and select the final scale(﻿s﻿). For clustered data, within-group dependency is investigated on the final scale(s). In the second step, the strength of the scale(s) is determined and further analyses are performed. The procedure was demonstrated on clustered item scores obtained from administering a questionnaire on quality of life in schools to 639 students nested in 30 classrooms.

**Conclusions:**

We developed a two-step, test-guided MSA for scale construction that takes into account sample fluctuation of all scalability coefficients and that can be applied to item scores obtained by a nonclustered or clustered sampling design.

**Supplementary Information:**

The online version contains supplementary material available at 10.1007/s11136-021-02840-2.

## Introduction

Nonparametric item response theory (NIRT) models [1, 2] are flexible measurement models that put relatively few restrictions on the data compared to parametric item response theory (PIRT) models, such as the Rasch model [3], the graded response model [4], and the partial credit model [5]. Therefore, NIRT models will fit the data relatively well. NIRT models have the attractive property that if the model fits the data, the sum score can be used to order respondents on the latent trait measured by the test (see [6] for details). Using the sum score for measurement is very common in quality of life research, but providing justification for using the sum score for measurement , for example, by showing that a NIRT model fits the data, is less common.

NIRT models have two major applications. First, NIRT models can be used as stand-alone measurement models. As NIRT models have relatively good fit compared to PIRT models, NIRT models are preferred for constructing tests and questionnaires that require ordering respondents, such as using the test scores to order respondents from high to low on the ability to cope independently, to select the 30% most capable respondents for a special training program, or to construct ordinal test norms such as percentile scores. Second, NIRT models can be used preliminary to PIRT models. Below, we discuss methods to identify items that do not fit the NIRT model and that should be removed from the test or questionnaire. As all popular PIRT models are special cases of NIRT models [7], the items selected for removal under the NIRT model will not fit under the PIRT model either. Removing these badly fitting items prior to PIRT analysis will simplify the PIRT analysis.

Mokken scale analysis (MSA; [1]; see also, e.g., [2, 8, 9]; and [10] for elaborate introductions to MSA and its methods) is a scaling method that consists of various tools to investigate the fit of NIRT models. The most popular tools of MSA are the *scalability coefficients* (or *H*-coefficients; [1], pp. 152–185), a diagnostic tool to evaluate whether the items form a scale, and the *automated item selection procedure* (AISP; Mokken, [1] pp. 190–194 ; see also Van Abswoude et al., [11]). The AISP selects items from a larger set of items into scales. Items that are not selected into a scale either violate the assumptions of the NIRT model, or have poor discriminatory power. In addition to the scalability coefficients and the AISP, MSA contains a set of methods to investigate whether the specific assumptions of the NIRT model holds. With regard to data collection, MSA assumes that the test scores are obtained using simple random sampling; that is, the respondents in the sample must not be clustered in groups, such as classes, hospitals, or geographical regions.

MSA is especially suitable for constructing quality of life (related) measures, as the NIRT properties are often sufficient for the intended use of the scales [12]. Recent examples of quality of life questionnaires that have been analyzed using MSA include the Heart disease-specific health-related quality of life (HeartQoL; [13]), the Participation and Activity Inventory for Children and Youth (PAI-CY; [14]), and the Rotterdam Diabetic Foot Study Test Battery (RDF [15]). In these studies, the data were collected using a simple random sample, yielding *nonclustered* test scores. A simple random sample is not always preferable in quality of life research, due to constraints in funding, time, or the sampling frame or due to a substantial preference for including multiple levels. In such cases, quality of life questionnaires are administered to respondents who are nested in groups, yielding *clustered* test scores (i.e., obtained by a cluster or multi-stage sampling design). Some authors who used such a sampling design include Elley et al. [16], who investigated health-related quality of life and related variables in 233 older patients from 42 general practitioners; Chen et al. [17], who investigated the quality of life of 1392 high-school and middle-school students nested in school classes; and Fisher and Li [18], who investigated the effects of a neighborhood walking program on quality of life among 182 older adults from 56 different neighborhoods in Portland, Oregon. In these examples, the interest is in measuring the trait of the patients, students, and older adults (level 1), whereas the grouping variables at level 2 (general practitioners, school classes, and neighborhoods) were considered to be a nuisance.

In this paper, we discuss two issues in MSA: First, point estimates, standard errors, and test statistics for scalability coefficients are unavailable for clustered data. Hence, currently scalability coefficients should not be used to evaluate clustered test scores. As the AISP also uses the point estimates and test statistics for scalability coefficients, the AISP should not be applied to clustered test scores either. Second, the AISP insufficiently takes into account sampling fluctuations , explained in detail hereunder. As a result, the AISP may be too liberal. We solved both issues by providing point estimates and standard errors for the scalability coefficients for clustered data and by incorporating a *z*-tests in a test-guided AISP (T-AISP) that tests all relevant hypotheses and that is available for both nonclustered and clustered test scores. We integrated the T-AISP into a comprehensive two-step, test-guided MSA, to guide the analysis for nonclustered and clustered data.

The remainder of this paper is organized as follows: First, we discuss the scalability coefficients and the AISP and elaborate on issues when using the scalability coefficients for clustered data and issues when using the AISP for both nonclustered and clustered data. Second, we propose solutions and introduce a T-AISP that tackles the issue pertaining to significance testing and the estimation methods for clustered data. Third, we incorporate the proposed methods in a two-step, test-guided MSA that can be applied to nonclustered and clustered data. Finally, using the two-step procedure, we analyzed data from the two-dimensional Dutch quality of life at school questionnaire Schaal Welbevinden met Docenten en Klasgenoten (Scale Well-Being with Teachers and Classmates) (SWMDK; [19]).

## Scalability coefficients

There are three types of scalability coefficients that can be used as a diagnostic tool for NIRT model fit and discriminatory power. Item-pair scalability coefficient $H_{ij}$ (for the pair of items *i* and *j*) is a normed correlation between the two items (e.g., [11]). Item-pair scalability coefficient $H_i$ is a normed item-rest correlation (i.e., the correlation between item *i* and the total score on the remaining items) and can be regarded as a discrimination index (e.g., [11, 20]). Total scale coefficient *H* is the weighted sum of the $H_i$s across all items, for which higher values indicate a more accurate ordering of respondents ([1], p. 152 ; [2], pp. 57–68). For a set of items $\text {min}(H_{ij})\le \text {min}(H_i)\le H \le \text {max}(H_i)\le \text {max}(H_{ij})$ ([2], p. 58).

Mokken ([1], pp. 184–185) called a set of items a scale (further referred to as a Mokken scale) if two criteria are met:

1 $$\begin{aligned}&H_{ij}>0 \text { for all item pairs;} \end{aligned}$$

2 $$\begin{aligned}&H_{i}\ge c \text { for all items,} \end{aligned}$$

with *c* being some positive lowerbound, for which $c=.3$ is often used. The first criterion is implied by the assumptions of Mokken’s NIRT models, which result in positive inter-item correlations. The second criterion is a practical requirement that ensures only sufficiently discriminating items are selected into the scale (see also [2], p. 68. Mokken [1], p. 185) provided benchmarks to determine the strength of a scale: $H\ge 0.5$ reflects a strong scale, $H\ge 0.4$ a medium scale, and $H\ge 0.3$ a weak scale. The stronger the scale, the more accurately persons can be ordered on the latent trait by means of their total score ([2], p. 68).

### Estimating scalability coefficients

So far, properties of scalability coefficients in the population were discussed. In research, we have finite samples, and scalability coefficients must be estimated from the sample data, where ${\widehat{H}}_{ij}$, ${\widehat{H}}_{i}$, and ${\widehat{H}}$ denote the estimate of population value $H_{ij}$, $H_i$, and *H*, respectively, and $SE_{{\widehat{H}}_{ij}}$, $SE_{{\widehat{H}}_{i}}$, and $SE_{{\widehat{H}}}$ denote the standard error of the estimate. For nonclustered data, Mokken ([1], p. 166; also, see [2], p. 49) derived estimates of scalability coefficients, and Kuijpers et al. [21] derived estimates for the standard errors. These estimation methods are referred to as *one-level methods*. For computational details, see the online Supplement Section 1 and 2. One-level methods assume the data are obtained from a simple random sample.

In clustered data, respondents are nested within groups, violating the assumption of an independent simple random sample that underlies the one-level methods. A typical aspect of clustered data is positive within-group dependency, in which test scores of respondents within the same group (or cluster) are more similar than test scores of respondents in different groups. A commonly used statistic to express within-group dependency is the intraclass correlation (ICC), which is the expected correlation between two test scores in the same group [22]. ICCs between 0 and 0.5 are common for measures of quality of life and related concepts (e.g., [16, 23–26]). In general, accounting for dependency in the data is advised if the ICC $>0$, as it can severely affect outcomes of statistical analyses [22, 27, 28]. For example, ignoring positive within-group dependency is a well-known cause of underestimated standard errors (e.g., [28, 29] although in rare cases overestimation may also happen, e.g., [30]). As a result, confidence intervals will be too narrow, making the estimates appear more precise than they actually are, and the type I error rates of significance tests will be inflated and should not be used.

For clustered data, estimates of scalability coefficients and standard errors are not yet available. However, Snijders [31] derived estimates for scalability coefficients for so-called *multi-rater data*, and Koopman et al. [32] derived estimates for their standard errors. Multi-rater data are also multilevel data, where level 2 (the subject level) is of primary interest, and level 1 (the rater level) is a nuisance. Snijders [31] proposed scalability coefficients for both levels: Within-rater scalability coefficients $H^W_{ij}$, $H^W_{i}$, and $H^W$ for level 1, and between-rater scalability coefficients $H^B_{ij}$, $H^B_{i}$, and $H^B$ for level 2. The estimates of the within-rater scalability coefficients and their standard errors may provide a viable alternative for Mokken’s scalability coefficients in clustered data.

### Hypothesis tests and confidence intervals for scalability coefficients

Mokken ([1], pp. 160–162) proposed a test of marginal independence to evaluate null hypotheses $H_{ij} = 0$, $H_i=0$ , or $H=0$. Let $S_{ij}$ denote the estimated covariance of item pair (*i*, *j*) and $S_i$ the estimated standard deviation of item *i*, in a sample of size *N*. Then, Mokken’s test statistic for item-pair coefficient $H_{ij}$ is defined as

3 $$\begin{aligned} \Delta _{ij}=\frac{S_{ij}}{S_i S_j} \sqrt{N-1}. \end{aligned}$$

Mokken’s test statistic is also available for item coefficients,

4 $$\begin{aligned} {\Delta }_i=\frac{\sum _{(j\ne i)} S_{ij}}{S_i \sum _{(j\ne i)} S_j} \sqrt{N-1}, \end{aligned}$$

and the total scale coefficient,

5 $$\begin{aligned} {\Delta }=\frac{\sum _i\sum _{(j>i)} S_{ij}}{\sum _i \sum _{(j>i)} S_i S_j} \sqrt{N-1} \end{aligned}$$

(see also [33], Sect. 3.5). Asymptotically, the test statistics follow a standard normal distribution Mokken ([1], pp. 162). A one-sided significance test can evaluate the null hypothesis $H_{ij}\le 0$ with alternative hypothesis $H_{ij}>0$. Let $z_\mathrm{crit}$ denote the critical value (i.e., the point on the normal distribution for a given significance level $\alpha$; e.g., $z_\mathrm{crit}\approx 1.645$ for $\alpha = .05$) that is compared to the test statistic to determine whether to reject the null hypothesis. The null hypothesis is rejected if the test statistic equals or exceeds $z_\mathrm{crit}$; for example, if $\Delta _{ij} \geq z_\mathrm{crit}$. This test assumes a simple random sample and is therefore only suited for nonclustered data.

Recently, Koopman et al. [34] (in press) defined a Wald-based significance test and a range-preserving significance test that both use the point estimates and standard errors of scalability coefficients to evaluate null hypotheses $H_{ij} = c$, $H_i=c$, or $H=c$, with *c* being some constant. The Wald-based test statistic for $H_{ij}$ is defined as

6 $$\begin{aligned} z_{ij} = \frac{{\widehat{H}}_{ij} - c}{SE_{{\widehat{H}}_{ij}}}. \end{aligned}$$

Let $g({\widehat{H}})$ denote a (logarithmic) transformation of ${\widehat{H}}$, with standard error $SE_{g({{\widehat{H}}})}$, and *g*(*c*) the transformation of hypothesized value *c* (for computational details see [34]). The range-preserving test statistic for $H_{ij}$ is defined as

7 $$\begin{aligned} z_{ij}^* = \frac{g({\widehat{H}}_{ij}) - g(c)}{SE_{g({\widehat{H}}_{ij})}}. \end{aligned}$$

The Wald-based and range-preserving test statistics are also available for item coefficients (denoted $z_i$ and $z_i^*$, by replacing ${\widehat{H}}_{ij}$ with ${\widehat{H}}_i$ in Eqs. (6) and (7), respectively) and the total scale coefficient (denoted *z* and $z^*$, by replacing ${\widehat{H}}_{ij}$ with ${\widehat{H}}$ in Eqs. (6) and (7), respectively; see also [34]). For nonclustered data, the range-preserving test has better type I error rates compared to the Wald-based test for very strong scales (e.g., $H>.7$; [34]).

As a result of the availability of both Wald-based and range-preserving test statistics, confidence intervals around the scalability coefficients can also be either Wald-based or range-preserving. Wald-based confidence intervals have the form

8 $$\begin{aligned} \text {CI} = {\widehat{H}} \pm 1.96 \times SE_{{\widehat{H}}} \end{aligned}$$

for a two-sided 95% confidence interval for the total scale coefficient ([1], p. 168; [32]). However, the maximum value of scalability coefficients is 1, and if *H* is close to 1 or its standard error is large, and Wald-based confidence intervals can include values larger than 1 and are biased due to a skewed sampling distribution. Hence, Koopman et al. [34] (in press) proposed range-preserving confidence interval . Let $g^{-1} \big [g({\widehat{H}})\big ]={\widehat{H}}$ denote the inverse of $g({\widehat{H}})$, then

9 $$\begin{aligned} \text {CI}^* = g^{-1}\big [g({\widehat{H}}) \pm 1.96 \times SE_{g({\widehat{H}})}\big ] \end{aligned}$$

is the two-sided 95% confidence interval of the total scale coefficient. This interval ensures all values to be within the possible range of the coefficient and has better coverage rates than the Wald-based interval in nonclustered data for high values of *H*.

## Automated item selection procedure

The objective of the AISP is to select as many items as possible into a scale, as long as these items meet the Mokken scale criteria (Eqs. 1 and 2). Table 1, upper panel, provides an overview of how the Mokken scale criteria currently are evaluated in the AISP. Criterion 1 is accepted if $\Delta _{ij} \geq z_\mathrm{crit}$ (Eq. 3), using null hypothesis $H_{ij}\le 0$ and alternative hypothesis $H_{ij}>0$. Criterion 2 is accepted if $\Delta _i \geq z_\mathrm{crit}$ (Eq. 4), using null hypothesis $H_{i}\le 0$ and alternative hypothesis $H_i>0$, and ${\widehat{H}}_i\ge c$. Hence, for Criterion 2, the hypothesis $H_i \ge c$ is not tested , but evaluated on the point estimate, which may render the procedure too liberal.

**Table 1:** Mokken Scale Criteria Evaluation by the AISP (upper panel) and the T-AISP (lower panel)

Criterion	AISP		
	Null hypothesis	Hypothesis matches criterion	Accepts criterion if
1: $H_{ij}>0$	$H_{ij}\le 0$	$\checkmark$	$\Delta _{ij} \geq z_\mathrm{crit}$
2: $H_{i}\ge c$	$H_{i}\le 0$	–	$\Delta _{i} \geq z_\mathrm{crit}$ and ${\widehat{H}}_i \ge c$

Criterion	T-AISP		
	Null hypothesis	Hypothesis matches criterion	Accepts criterion if
1: $H_{ij}>0$	$H_{ij}\le 0$	$\checkmark$	$z_{ij} \geq z_\mathrm{crit}$
2: $H_{i} > c$	$H_{i}\le c$	$\checkmark$	$z_{i} \geq z_\mathrm{crit}$

The AISP starts with a (typically large) set of items that have been administered to a sample of respondents, so for each item in the set, item scores are available. The AISP uses the following algorithm.

1. *Select the first two items in the scale* These two items have the highest value of ${\widehat{H}}_{ij}$ and both Mokken scale criteria must have been accepted. If for no item-pair both Mokken scale criteria are accepted, no items are selected and the AISP stops.
2. *Select the next items into the scale* The next item selected in the scale is the item for which both Mokken scale criteria are accepted and that produces the highest ${\widehat{H}}$-value when computed on all selected items. Step 2 is repeated until there are either no more items left, or until no more items can be added for which both Mokken scale criteria are accepted.
3. *Start the next scale* The AISP returns to Step 1 to form a next scale using only the unselected items. If there are no more items left or if there are no more pairs of items for which the Mokken scale criteria are accepted, the AISP stops.

Note that the value $z_\mathrm{crit}$ is adjusted in each subsequent step using a Bonferroni correction for the number of tests performed in the previous steps and the current step of the algorithm ([2] , p. 72). Alternative algorithms for automated item selection in MSA have been suggested (e.g., [35–37]), but are not discussed.

The AISP can be applied using different values of lowerbound *c*. By increasing *c*, the criterion for item scalability becomes more stringent. Stringent criteria lead to shorter scales with higher discrimination power in the sample under investigation. When applying the AISP to investigate whether a set of items form one or more Mokken scales, [38] (see also [8]) advised to use increasing values for *c* (e.g., $c = 0, .05, .10, \ldots , .55$). Typically, for a unidimensional scale satisfying the NIRT model, for small values of *c*, all or most items are in a single large scale, as *c* increases most items are in a single smaller scale and the remaining items are unscalable, and as *c* increases further, there are only one or a few small scales and several unscalable items remain. For a multidimensional scale satisfying the NIRT model, typically for small *values of c*, all or most items are in one large scale, as *c* increases most or all items are divided over two or more scales, and as *c* increases further, there are two or more smaller scales and several unscalable items. Note that the number of Mokken scales does not necessarily reflect the dimensionality of the item set, this depends on the correlation between the dimensions and the level of item discrimination [39].

Because $H_i > c$ is not tested and only point estimates are used for the evaluation of Criterion 2, the sampling fluctuation is not taken into account and the item selection is too liberal. Ignoring sample fluctuation can result in the inclusion of items that do not contribute to (or possibly negatively affect) accurate measurement of the scale in the population. Hence, we require an alternative significance test in the AISP that meets the following requirements: It can test null hypotheses for values of *c* other than zero and is available for both nonclustered and clustered data.

## Solving the two MSA issues

### Point estimates , standard errors , and statistical tests of scalability coefficients for clustered data

As noted earlier, no point estimates or standard errors are available for scalability coefficients for clustered data, but for multi-rater data, point estimates [31] and standard errors [32] have been derived. For clustered data, we derived point estimates and standard errors in the Online Supplement by slightly modifying the point estimates and standard errors for multi-rater data, which we coin the *two-level methods*. It turned out that the point estimates of the two-level method are equivalent to the point estimates of the one-level method, whereas the standard errors account for the additional variation that is typical for clustered data. For computational details and explanation of the modification, we refer to the Online Supplement, Sections 1 and 2. The estimates based on the two-level method can be plugged into Eqs. (6), (7), (8), and (9) to get the test statistics and confidence intervals of scalability coefficients for clustered data.

In a small-scale simulation study (Online Supplement, Section 3), we compared one-level methods to two-level methods, and Wald-based methods to range-preserving methods for Mokken’s scalability coefficients in clustered data. Point estimates of the scalability coefficients were accurately estimated in all conditions. In general, estimating standard errors and confidence intervals using the two-level method produced less bias and better coverage rates than using the one-level method. This was especially true for larger ICC levels and for larger groups. For small ICC values (i.e., ICC $\le$ 0.12), especially for small group sizes (i.e., below 10), the standard errors estimated using the two-level method were slightly conservative, but the absolute difference was small compared to the one-level method. Hence, we recommend to use the two-level method for estimating standard errors in clustered data. The coverages of the Wald-based and range-preserving confidence intervals were approximately similar, but the Wald-based method was slightly more symmetric. As in practice, scalability coefficients are seldom very close to 1, and all relevant hypotheses regarding scalability coefficients are in the range from 0 to 0.55, we believe the additional value of range-preserving confidence intervals in practice is limited. Hence, we recommend Wald-based confidence intervals and significance tests.

### Test-guided automated item selection procedure

As noted earlier, both Criterion 1 and Criterion 2 (Eqs. 1 and 2) should be tested in the AISP, to gain confidence that the formed scales satisfy both Mokken scale criteria. In addition, the testing procedure should be available for both nonclustered and clustered data. We propose a test-guided AISP (T-AISP) that uses the same algorithm of the AISP, but that evaluates the criteria of a Mokken scale using different significance tests. Specifically, in the T-AISP, Mokken’s $\Delta _{ij}$ and $\Delta _i$ are replaced by the Wald-based test statistics $z_{ij}$ (Eq. 6), with null hypothesis $H_{ij}\le 0$ and alternative hypothesis $H_{ij}>0$, and $z_i$ (cf. Eq. 6), with null hypothesis $H_i\le c$ and alternative hypothesis $H_{i}>c$, respectively. Table 1, lower panel, provides an overview of how the Mokken scale criteria are evaluated in the T-AISP. Criterion 1 is accepted if $z_{ij} \geq z_\mathrm{crit}$. Criterion 2 is accepted if $z_i \geq z_\mathrm{crit}$. Consequently, the evaluation of Criterion 2 in the AISP that uses the point estimate, ${\widehat{H}}_i>c$ (Table 1, third column in upper panel), is redundant and can be removed from the algorithm. Although the original AISP is also partially test-guided (Criterion 1 is tested), we refer to the adjusted evaluation as the test-guided procedure, because only in the T-AISP algorithm, both criteria of a Mokken scale are tested. We distinguish between a T-AISP using one-level methods and a T-AISP using two-level methods. The T-AISP using one-level methods computes the standard errors in $z_{ij}$ and $z_i$ using the one-level methods, which gives an appropriate test for nonclustered data. The T-AISP using two-level methods computes these standard errors using the two-level methods, which gives an appropriate test for clustered data. Using the Wald-based significance tests in the T-AISP results in a slightly different second Mokken scale criterion compared to the original definition: A scale for which $H_i>c$ for all items, rather than $H_i\ge c$. Note that replacing $\Delta _{ij}$ and $\Delta _{i}$ in Table 1, upper panel, by $z_{ij}$ and $z_{i}$, respectively, while retaining the same hypotheses, makes the AISP available for clustered data when two-level methods are used, but retains the issue that Criterion 2 is not tested.

The major difference of the T-AISP compared to the AISP is that Criterion 2 of a Mokken scale (Eq. 2) is statistically tested, rather than evaluated using a point estimate. As a result, T-AISP is more conservative compared to the AISP, because uncertainty of ${\widehat{H}}_i$ is taken into account. As the point estimates become more accurate (e.g., for larger samples), the uncertainty becomes smaller, and the formed scales by the T-AISP approach those of the AISP more closely. When there is a substantial amount uncertainty, the T-AISP will generally result in more and smaller scales and is likely to show more unscalable items compared to the AISP for the same lowerbound *c*. Note that the resulting scale patterns in the T-AISP can be different from patterns that emerge by using more stringent criteria for lowerbound *c* in the AISP, because uncertainty can differ across items, samples, and sample sizes, whereas a given *c* is fixed regardless of the items and sample.

## A two-step, test-guided MSA, for nonclustered and clustered data

**Fig. 1:**
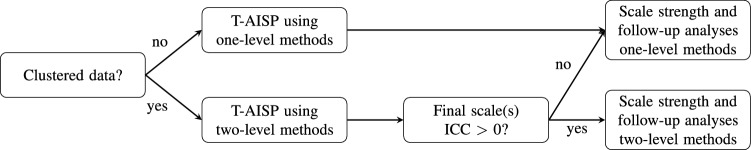
Flow chart of the two-step test-guided procedure for scale construction

The two-step, test-guided MSA is a procedure to create scales from a set of items, evaluate the strength of these scales, and perform follow-up analyses such as fit diagnostics of the NIRT models and possibly PIRT models. Figure 1 shows a flow chart of the procedure, which is elaborated on below.

The procedure commences with determining whether the data are clustered, based on the sampling design. Hence, if data were collected using a simple random sampling design, the data are nonclustered, whereas if a (two-stage) cluster sampling design was used, the data are clustered. For clustered data, we discourage investigating the ICC of a test score at this stage. The rationale is that test scores in this stage are based on a set of items for which the quality is unsure, as the goal of MSA is forming good-quality scales, and poor items should not affect decisions based on the ICC. For example, unscalable items may mask within-group dependency that is possibly present when using the final scale. If one was to perform the T-AISP using one-level methods, the one-level standard errors of large-ICC items are likely to be substantially too small, and there is an increased risk of incorrectly admitting these items to the final scale.

In Step 1, the T-AISP is applied to form one or more Mokken scales. The significance tests in the algorithm are performed using one-level methods for nonclustered data and two-level methods for clustered data. The T-AISP is performed using increasing lowerbounds from 0 to .55 in steps of .05 to investigate how scales are formed and how stable they are, although the number and range of lowerbounds may be adjusted based on practical or theoretical considerations. Final scales are selected based on stability, discrimination, and possibly theoretical considerations.

For clustered data, the within-group dependency of the formed scales is evaluated. The within-group dependency of each scale is investigated by estimating the ICC of the total score per scale and performing an *F* test to test the null hypothesis that the ICC is zero (for computational details, see [40], pp. 19–23). This is the *F* test also used in analysis of variance and assumes that the test scores within a group are normally distributed. If the *F* test is not significant, it is plausible that the ICC is zero, and accounting for the nesting is not necessary in the subsequent statistical analyses. However, if the ICC is significantly larger than zero, the subsequent analyses should be estimated using multilevel data analysis methods.

Step 2 is determining the strength of the final scales and performing follow-up analyses, using one-level methods for nonclustered data and for clustered data without within-group dependency, and two-level methods for clustered data with significant within-group dependency. The strength of the scale is evaluated using a 95% Wald-based confidence interval around total scale coefficient estimate ${\widehat{H}}$ (Eq. 8) to get plausible values of the population coefficient *H*. The fit of Mokken models is investigated using several available methods, such as conditional association and manifest monotonicity (e.g., [8]). Items that show (severe) misfit may be adjusted or removed [41]. Subsequently, reliability analysis may be performed or more strict measurement models (e.g., PIRT models) may be fitted.

## Real-data example

The SWMDK is a two-dimensional scale designed to measure a student’s perception of their well-being at school with teachers and classmates. The items are scored on a five-point Likert scale, ranging from 1 (*not true at all*) to 5 (*completely true*), and contain elements of relationships, interactions, and feelings towards the teachers and classmates. The SWMDK consists of seven items pertaining teachers (SWMD , items 1 to 7) and six items pertaining classmates (SWMK, items 8 to 13 ;[42]), although shorter versions have been used (e.g., [43, 44]). To our knowledge, no scalability analysis has been performed on this scale, and therefore, it is unsure whether the items may be used in one scale, whether subscales should be formed, and which items should be included in the scale(s). For the SWMD, Cronbach’s $\alpha$s varied between 0.63 and 0.84 and for the SWMK between 0.68 and 0.83 [42–44]. Table 2 shows the 13 items of the SWMDK.

**Table 2:** Item content, mean, and standard deviation for each item and for the total scale of the SWMDK

Item		*M*	SD
1	The teachers usually know how I feel	2.84	0.89
2	I can talk about problems with the teachers	3.18	0.92
3	If I feel unhappy, I can talk to the teachers about it	3.03	0.98
4	I feel at ease with the teachers	3.52	0.77
5	The teachers understand me	3.23	0.81
6	I have good contact with the teachers	3.34	0.83
7	I would prefer to have other teachers*	3.22	0.85
8	I have a lot of contact with my classmates	4.06	0.76
9	I would prefer to be in another class*	3.89	1.08
10	We have a nice class	3.89	0.96
11	I get along well with my classmates	4.01	0.73
12	I sometimes feel alone in the class*	4.12	0.92
13	I enjoy hanging out with my classmates	4.00	0.74
Total scale		3.57	0.53

*SWMDK* Schaal Welbevinden Met Docenten en Klasgenoten. The items were translated from Dutch. For the original items, see pp. 79–83 in Zijsling et al. [42]. *M* mean, *SD* standard deviation. Items 1 to 7 pertain teachers, items 8 to 13 pertain classmates

*Reversely scored item that has been recoded

### Method

The data were collected in 814 classes at 94 secondary schools in the Netherlands, as part of a large-scale cohort study COOL$^{5-18}$ (see [42], for sampling and consent procedures). The data used in this analysis consisted of a subset of 30 classes (each from a different school) to better reflect everyday practice in quality of life research in which smaller samples are more common. The average class size was 21.30 (SD = 6.49), in a total sample of 639 students. Table 2 shows the means and standard deviations of the items and the total scale of the SWMDK.

**Fig. 2:**
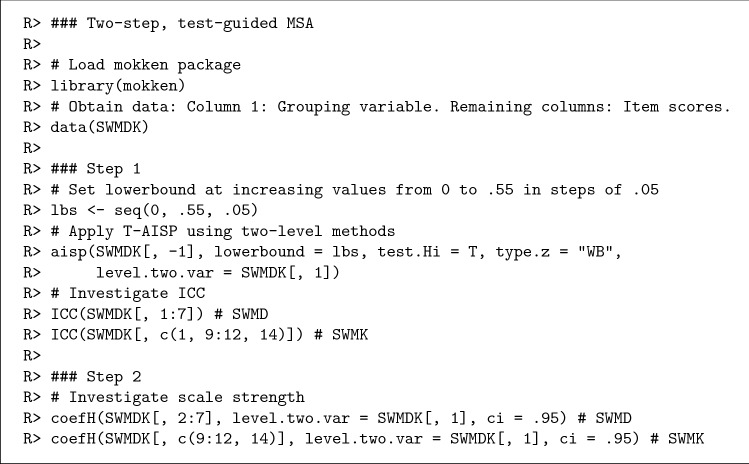
R syntax to obtain the main results of the two-step, test-guided MSA in the real-data example. R> denotes the R prompt and # precedes a comment

The data were collected in classes by means of a cluster sampling design, resulting in clustered data. Hence, we performed the two-step, test-guided MSA instructions for clustered data to investigate the scalability of the SWMDK. We desired a stable set or sets of items that was sufficiently discriminative, preferring Mokken scales with $H_i\ge 0.3$ for all items. All analyses were conducted in R [45] using the R package mokken [46, 47], which also contains the data. The R syntax to obtain the results in this section are available to download from the Open Science Framework: http://osf.io/y7xud. The R syntax in Fig. 2 is a shortened version.

### Results

In Step 1 of the two-step, test-guided MSA, for lowerbounds $c=0.00$ and 0.05, all items were included in one scale (Table 3, columns 1 and 2), implying that the first Mokken scale criterion ($H_{ij}>0$) was accepted. For $c=0.15$ to 0.45, two scales were formed, one predominantly containing SWMD items and one predominantly containing SWMK items. Items 7 and 12 were not included for $c\ge 0.25$ and item 8 was not included for $c=0.45$ (Table 3, columns 3 to 10). For $c\ge 0.50$, the items fell apart into three small subscales and several unscalable items (Table 3, columns 11 and 12). This reflects the typical pattern expected for a two-dimensional scale. The results supported the division into two separate scales: The first well-being with teachers and the second well-being with classmates. Items 7 and 12 were removed from the final scales as they contributed too little to accurate measurement of the final scales ($c < 0.25$); hence, they did not meet the second Mokken scale criterion (which we defined as $H_i>0.3$ for all items). Apparently items 7 and 12 contain elements that either are reflective of another construct, or misunderstood by the students. We continued with the procedure using the six-item SWMD and the five-item SWMK.

**Table 3:** Scales formed by the T-AISP for increasing values of lowerbound *c*

Item	*c*											
	0.00	0.05	0.10	0.15	0.20	0.25	0.30	0.35	0.40	0.45	0.50	0.55
1	1	1	1	1	1	1	1	1	1	1	1	0
2	1	1	1	1	1	1	1	1	1	1	1	1
3	1	1	1	1	1	1	1	1	1	1	1	1
4	1	1	1	1	1	1	1	1	1	1	1	2
5	1	1	1	1	1	1	1	1	1	1	1	2
6	1	1	1	1	1	1	1	1	1	1	0	0
7	1	1	1	1	1	0	0	0	0	0	0	0
8	1	1	1	2	2	2	2	2	2	0	3	0
9	1	1	1	2	2	2	2	2	2	2	2	3
10	1	1	1	2	2	2	2	2	2	2	2	3
11	1	1	1	2	2	2	2	2	2	2	3	0
12	1	1	0	2	2	0	0	0	0	0	0	0
13	1	1	1	2	2	2	2	2	2	2	2	0

The number in each cell represents the scale to which the item was assigned. Unscalable items are denoted by 0

The average test score for the final, six-item SWMD was 3.20 (SD$=0.68$) and of the final, five-item SWMK 3.97 (SD$=0.68$). Table 4 shows the ICC per item and for the final scales. The ICC fluctuated across the items, indicating that some items have a larger group effect than others. The estimated ICC of the SWMD was .169 and was significantly larger than zero ($F(29, 609) = 5.31$, $p < 0.001$). The estimated ICC of the SWMK was .183 and was also significantly larger than zero ($F(29, 609) = 5.75$, $p < 0.001$). Hence, we continue the analyses using two-level methods.

**Table 4:** Scalability coefficients, standard errors, and wald-based confidence intervals estimated using the Two-level method, and ICCs for each item and the total scale of the SWMD and the SWMK

SWMD					SWMK				
Item	${\widehat{H}}$	SE	95% CI	ICC	Item	${\widehat{H}}$	SE	95% CI	ICC
1	0.609	0.033	[0.545; 0.674]	0.120	8	0.547	0.036	[0.477; 0.617]	0.077
2	0.641	0.026	[0.589; 0.693]	0.111	9	0.551	0.036	[0.480; 0.621]	0.103
3	0.619	0.029	[0.562; 0.676]	0.103	10	0.644	0.025	[0.595; 0.693]	0.196
4	0.634	0.033	[0.568; 0.699]	0.142	11	0.594	0.031	[0.532; 0.655]	0.111
5	0.650	0.028	[0.594; 0.705]	0.082	12	–	–	–	–
6	0.566	0.031	[0.506; 0.626]	0.129	13	0.627	0.028	[0.572; 0.682]	0.097
7	–	–	–	–					
Total	0.620	0.026	[0.570; 0.670]	0.169	Total	0.592	0.025	[0.543; 0.642]	0.183

${\widehat{H}}$ estimated scalability coefficient, *SE* standard error, *CI* confidence interval, *ICC* intraclass correlation

In Step 2, the SWMD and the SWMK were evaluated as strong scales, as both (two-level) 95% confidence intervals exceeded the threshold of 0.5 for a strong scale (see Table 4, bottom row). For completeness, the point estimates, standard errors, and confidence intervals of the item coefficients for both scales are shown in Table 4. Given that Mokken’s NIRT model holds, the scales could be used to order respondents using the sum scores on the scales, with all related (ordinal) measurement properties.

We now illustrate what would have happened if the traditional AISP would have been used that does not take into account sampling fluctuation and the nested data structure. Results showed that for $c\le 0.2$ all items were included in one scale. For $c=0.25$ , item 12 was dropped from the one scale. For $c\ge 0.3$ , two scales were formed, one containing SWMD items (not including item 7 for $c\ge 0.4$) and the other containing SWMK items (not including item 12 for $c\ge 0.4$ and also item 8 for $c=0.55$).

Comparing the results of the AISP to the results of the T-AISP demonstrates that, for this data example, similar scale patterns emerged across different values of the lowerbound. However, the AISP retained more items in less scales for larger lowerbounds. In addition, for $c=0.3$ (the lowerbound that is often used as default), the AISP divided all items into two subscales, whereas the T-AISP considered items 7 and 12 unscalable. These differences are a consequence of the AISP not taking uncertainty of $H_i$ into account, and this uncertainty is quite substantial in this small data example.

## Discussion

This paper introduced two major advancements in MSA: First, point estimates and standard errors for scalability coefficients were derived for clustered data (where respondents are nested in groups). Until now, estimating scalability coefficients and their standard errors were unavailable for clustered data. However, point estimates and standard errors for within-rater scalability coefficients , which are similar in interpretation as the original scalability coefficients, were available for multi-rater data [32], data that also have a two-level structure. We proposed a slight adaptation of the estimates that resulted in two-level methods, for which the point estimates are identical to the traditional (one-level) point estimates for scalability coefficients, but for which the standard errors are accurate and have little bias in clustered data. To keep the paper readable, details of the estimation, accuracy, and bias of the point estimates and standard errors have been diverted to the Online Supplement.

Second, a test-guided automated item selection procedure (T-AISP) was introduced. The traditional AISP tested only Criterion 1 of a Mokken scale ($H_{ij}>0$), and evaluated Criterion 2 ($H_i>c$) by testing whether $H_i>0$ and checking whether the point estimate ${\widehat{H}}_i\ge c$. By implementing a Wald-based test statistic in the T-AISP, we enabled the direct testing of both criteria of a Mokken scale. In addition, by using the newly developed standard errors based on the two-level method, the T-AISP could also be adapted to clustered data. As illustrated by a real-data example, the T-AISP is more conservative than the AISP. So when using the T-AISP, a researcher may expect that less items will end up in the scale. In addition, especially for large sets of items, the computation time of the T-AISP may be considerably longer than the AISP. In future research, simulation studies using population covariance structures may show whether the T-AISP is indeed a better item selection procedure than the AISP. A possible alternative for the Wald-based test statistic in the T-AISP would be the marginal modeling approach of Van der Ark et al. [33] for flexible and distribution-free testing of scalability coefficients. However, their test could only be applied to a limited number of dichotomous items. The comparison of their test to our test was beyond the scope of this paper, but could be a topic of future research.

We integrated the two advancements into a two-step, test-guided MSA for scale construction, which is available for nonclustered and clustered data. The first step is performing a T-AISP and select final scale(s) with items that meet specified scalability criteria, using one-level methods for nonclustered and two-level methods for clustered data. For clustered data, the ICC is estimated on the final scale(s) and an F test is performed to test whether the ICC is significantly larger than zero. In the second step, the strength of the scale(s) is determined using 95% Wald-based confidence intervals and further analyses are performed, using one-level methods for nonclustered data and for clustered data without within-group dependency, and two-level methods for clustered data with within-group dependency.

We applied the two-step, test-guided MSA to the 13-item scale SWMDK, intended to measure students’ well-being at school with teachers and classmates. As data were collected using a cluster sampling design, two-level MSA methods were necessary. The T-AISP resulted in two reduced subscales (the 6-item SWMD and 5-item SWMK), both with significant within-group dependency. In the second step, both scales were evaluated as strong scales. Note that the conclusions on the scalability were based on a subset of respondents from a larger dataset; when the procedure was applied to the original dataset, two subscales were formed as well, but for each scale, all items remained, resulting in a seven-item SWMD (evaluated as a medium to strong scale) and a six-item SWMK (evaluated as a strong scale), both with significant within-group dependency.

The next step in making MSA available for clustered data is generalizing the methods to investigate NIRT model assumptions monotonicity, local independence and non intersection, as these are critical for the implied measurement properties of Mokken’s NIRT models. In addition, our proposed procedure can only handle two-level data. The procedure would benefit from a generalization to more complex sampling designs such as a three-level nested sample, for example, when students are nested in classrooms, nested in schools, or a cross-nested sample, where respondents are nested in more than one group.

## Supplementary Information

Below is the link to the electronic supplementary material.

Supplementary file 1 (PDF 217 kb)

## Data Availability

The data are available in R package mokken, and the other material and code are available to download from the Open Science Framework: http://osf.io/y7xud.
