# Supplementary material for: A two-step, test-guided Mokken scale analysis, for nonclustered and clustered data
Source: Qual Life Res. 2021 May 13;31(1):25–36. doi: 10.1007/s11136-021-02840-2 (PMC8800881; doi:10.1007/s11136-021-02840-2)
Supplement: Supplementary file 1 — (PDF 217 kb) [file 11136_2021_2840_MOESM1_ESM.pdf]

Supplement to "A Two-Step, Test-Guided Mokken Scale Analysis, for Nonclustered and Clustered data"

This document contains the technical details pertaining the estimation of item-score probabilities and scalability coefficients in nonclustered and clustered data, using different methods of computing item-score proportions. Also, we give results from a simulation study in which the estimation methods were investigated.

1 Estimating Item-Score Probabilities

Let a test consist of I dichotomous items, indexed i or j $(i, j = 1, 2, \ldots, I; i \neq j)$, which is administered to S groups, indexed s $(s = 1, 2, \ldots, S)$, with R_s respondents in group s, indexed r $(r = 1, 2, \ldots, R_s)$. For nonclustered data, $S = 1$ and R_s is the total number of respondents. Let X_{sri} denote the score of group s by respondent r on item i, with realization x_{sri} ($x_{sri} = 0$ or 1). Let $P(X_{sri} = 1)$ denote the univariate item-score probability of endorsing item i for a randomly selected respondent, and $P(X_{sri} = 0, X_{srj} = 1)$ denote the bivariate item-score probability of endorsing item j, but not item i. For dichotomous items $P(X_{sri} = 0) = 1 - P(X_{sri} = 1)$. There are 2^I item-score patterns $\mathbf{x}_{1\,2\ldots I}^{x_1 x_2 \ldots x_I} = (X_{sr1} = x_1, X_{sr2} = x_2, \ldots, X_{srI} = x_I)$. Let probability $P(\mathbf{X} = \mathbf{x}_{1\,2\ldots I}^{x_1 x_2 \ldots x_I}) = P(X_{sr1} = x_1, X_{sr2} = x_2, \ldots, X_{srI} = x_I)$ denote the probability for scoring item-score pattern $\mathbf{x}_{1\,2\ldots I}^{x_1 x_2 \ldots x_I}$. The item-score pattern probabilities are collected in vector $\mathbf{p}$.

In nonclustered data item-score proportions are computed as

$$\widehat{P}(X_{sri} = 1) = \frac{1}{\sum_{s=1}^{S} R_s} \sum_{s=1}^{S} \sum_{r=1}^{R_s} x_{sri}, \tag{1}$$

$$\widehat{P}(X_{sri} = 0, X_{srj} = 1) = \frac{1}{\sum_{s=1}^{S} R_s} \sum_{s=1}^{S} \sum_{r=1}^{R_s} (1 - x_{sri}) x_{srj}, \tag{2}$$

and

$$\widehat{P}(\mathbf{X} = \mathbf{x}_{1\,2\ldots I}^{x_1 x_2 \ldots x_I}) = \frac{1}{\sum_{s=1}^{S} R_s} \sum_{s=1}^{S} \sum_{r=1}^{R_s} (x_{sr1})^{x_1} (x_{sr2})^{x_2} \ldots (x_{srI})^{x_I}, \tag{3}$$

which amounts to averaging the frequencies across all respondents. Note that value S and subscript s both equal 1 in nonclustered data.

For clustered data, Snijders (2001) argued that using the proportions in Equations 1 to 3 may be biased estimators for the probabilities when the group size R_s is related to the latent trait value of the group. To avoid biased estimates, he suggested to compute proportions as

$$\widehat{P}(X_{sri}=1)=\frac{1}{S}\sum_{s=1}^{S}\frac{1}{R_s}\sum_{r=1}^{R_s}x_{sri}, \tag{4}$$

$$\widehat{P}(X_{sri}=0,X_{srj}=1)=\frac{1}{S}\sum_{s=1}^{S}\frac{1}{R_s}\sum_{r=1}^{R_s}(1-x_{sri})x_{srj}, \tag{5}$$

and

$$\widehat{P}(\mathbf{X}=\mathbf{x}_{1\,2\,\ldots\,I}^{x_1x_2\ldots x_I})=\frac{1}{S}\sum_{s=1}^{S}\frac{1}{R_s}\sum_{r=1}^{R_s}x_{sr1}^{x_1}x_{sr2}^{x_2}\ldots x_{srI}^{x_I} \tag{6}$$

(see also Section 3.2 in Koopman et al., 2017). These computations amount to first averaging the frequencies per group into group proportions, and then averaging these group proportions across groups. Note that when group sizes are equal, there is no difference between averaging frequencies across all respondents (Eq. 1 to 3) and averaging group proportions (Eq. 4 to 6).

2 Computing Scalability Coefficients and Standard Errors

On population level, Mokken's scalability coefficients and Snijders' within-rater scalability coefficients are identical, hence we refer to them as H_{ij} for item-pair coefficients, H_i for item coefficients, and H for the total-scale coefficient. Let an (dichotomous) item set be ordered in descending popularity (or ascending difficulty) such that

$$P(X_{sr1}=1)\geq P(X_{sr2}=1)\geq\ldots\geq P(X_{srI}=1). \tag{7}$$

Hence, item 1 is most popular (least difficult) and item I least popular (most difficult). For a item set with $i<j$, item-pair scalability coefficient H_{ij} is defined as

$$H_{ij}=1-\frac{P(X_{sri}=0,X_{srj}=1)}{P(X_{sri}=0)P(X_{srj}=1)}, \tag{8}$$

the item scalability coefficient H_i as

$$H_i = \frac{\sum_{j \neq i} P(X_{sri} = 0, X_{srj} = 1)}{\sum_{j \neq i} P(X_{sri} = 0) P(X_{srj} = 1)}, \tag{9}$$

and the total-scale coefficient H as

$$H = \frac{\sum_i \sum_{j>i} P(X_{sri} = 0, X_{srj} = 1)}{\sum_i \sum_{j>i} P(X_{sri} = 0) P(X_{srj} = 1)} \tag{10}$$

(for polytomous generalizations, see, e.g., Molenaar, 1991; Koopman, Zijlstra, & Van der Ark, 2020). Mokken's scalability coefficients are estimated in data samples by replacing the probabilities $P(X_{sri} = 1)$, $P(X_{sri} = 0)$, and $P(X_{sri} = 0, X_{srj} = 1)$ by sample proportions in Equation 1 and 2, respectively, whereas Snijders' within-rater scalability coefficients are estimated by replacing the probabilities by the sample proportions in Equation 4 and 5, respectively. Because there exist no difference between Mokken's coefficients and Snijders' within-rater coefficients, the estimation methods of within-rater coefficients may be viable to estimate Mokken's coefficients in clustered data.

For nonclustered data, standard errors of scalability coefficients were derived by assuming that the item-score pattern frequencies follow a multinomial distribution with parameters $\mathbf{p}$ and R_s, estimated using Equation 3 (Kuijpers et al., 2013). Bias of the point estimates and standard errors in nonclustered data was negligible (Kuijpers et al., 2016). For multi-rater data, standard errors were derived by assuming that the item-score pattern frequencies follow a multinomial distribution per group with parameters $\mathbf{p}$ and R_s, accounting for overdispersion in the data (Koopman, Zijlstra, & Van der Ark, 2020). Item-score probabilities in $\mathbf{p}$ are estimated using Equation 6. Koopman, Zijlstra, De Rooij, and Van der Ark (2020) showed that bias of the standard errors was generally negligible, except when group sizes differed. This may be explained because averaged group proportions from Equation 6 were used to estimate the standard errors, in which small groups weigh as heavily as large groups, even though they are usually less accurate. This can introduce relatively much noise, leading to overestimation of the standard error. For clustered data, we therefore propose replacing Equations 4 and 5 with Equations 1 and 2 to estimate scalability coefficients in Equations 8 to 10, and replacing

Equation 5 with Equation 2 to compute **p** for the multinomial distribution for the standard errors in clustered data. Hence, we assume that there is no relation between the latent trait value of the group and the group size. We will refer to the estimation method for nonclustered data as the *one-level method* and to our adapted estimation method for clustered data as the *two-level method*. The point estimates for the one- and two-level methods are identical, but their standard errors differ.

3 Simulation Study

To determine whether two-level estimates for the scalability coefficients and standard errors outperform the one-level methods in clustered data, we need to investigate their performance. Because the one- and two-level point estimates are identical, we omit the level and refer to them as the point estimate. The performance of the point estimate, the one- and two-level standard error, and the one- and two-level Wald-based and range-preserving confidence intervals were investigated in a Monte Carlo simulation study (see, e.g., Morris et al., 2019). This numerical computer study involves repeatedly creating data by random sampling. Because the true population model and parameter value is known in such a study, the behavior of the estimated statistic of interest can be evaluated under specific conditions, such as in small samples. Syntax files are available to download from the Open Science Framework (OSF): `http://osf.io/y7xud`.

Method

Data were generated using a graded response model (Samejima, 1969), an item response model for polytomous data that is a special case of Mokken's NIRT model (Van der Ark, 2001). Each respondent has a latent trait value $\theta_{sr} = \theta_s + \delta_{sr}$, which combines a group component with a respondent-specific (individual) component. Each simulated data set consisted of S groups, with R_s respondents in group s, and item scores of seven 5-category items.

Design Factors. The data-generation mechanism varied in terms of number of groups (S), group size (R_s), and the degree of within-group dependency as denoted by the ICC.

Number of Groups. The levels for the number of groups S were 10, 30, 50, and 100 groups, where it is expected that $S = 10$ is too small to result in accurate estimates (e.g., Snijders & Bosker, 2012, p. 48).

Group Size. Group size R_s had eight levels. Six levels had equal group size (i.e., 2, 5, 10, 20, 50, and 100 respondents per group) and two levels had unequal group size sampled from a discrete uniform distribution defined over the interval $[10,30]$. For the levels with unequal group sizes, the group size was either independent of the group trait value θ_s or the group sizes were matched to the θ_s values, so that a larger group size implied a higher θ_s value (discussed in detail below).

Within-Group Dependency. The ICC had five levels: very small (.06), small (.13), medium (.25), large (.47), and very large (.69). The ICC was manipulated via the variance of the respondent-specific component δ_{sr}, denoted σ^2_δ. The values were $\sigma^2_\delta = .93, .85, .70, .45$, and .20 for the five ICC levels, respectively, and was computed for each level by using a large sample ($S = 100,000, R_s = 10$). The variance of the group component θ_s was set to $\sigma^2_\theta = 1 - \sigma^2_\delta$, so the variance of the combined trait θ_{sr}, $\sigma^2_{\theta_{sr}} = \sigma^2_\theta + \sigma^2_\delta = 1 - \sigma^2_\delta + \sigma^2_\delta = 1$ for each level, resulting in an identical population value for all conditions. For larger σ^2_δ values, the individual effect in the latent trait θ_{sr} is larger (and the group effect smaller), making item scores within the same group less similar, and as a result, test scores within a group are less similar and the ICC of the test score is lower.

All levels were fully crossed, which resulted in $8 \times 8 \times 5 = 160$ conditions. Per condition, $Q = 1000$ datasets were generated, indexed by q ($q = 1, 2, \ldots, Q$). The population value of H was .515 for all conditions, determined using a large sample ($S = 1,000,000$). The empirical standard error SE_H was computed for each condition as the standard deviation of $\widehat{H}$ across the Q replications, and varied between .005 and .127.

Performance Measures. For the simulated dataset in each replication, we computed the point estimate of the total-scale scalability coefficient $\widehat{H}_q$, its standard error SE_q, the 95% Wald-based confidence interval CI_q, and the 95% range-preserving confidence interval CI^*_q, using the one- and two-level methods. Population value H is computed using a large sample ($S = 1,000,000$) and its standard error SE_H as the standard deviation of $\widehat{H}$ across the replications within a condition. Performance measures were bias of $\widehat{H} = Q^{-1} \sum_{q=1}^{Q} (\widehat{H}_q - H)$, bias of $SE_{\widehat{H}} = Q^{-1} \sum_{q=1}^{Q} (SE_q - SE_H)$, and coverage of the confidence interval (proportion of times H is

included in CI_q or CI_q^*). Also, we compared the symmetry of the undercoverage of the confidence interval for both type of intervals; that is, whether the undercoverage was equally distributed on both sides of the interval. A satisfactory coverage (i.e., .95) with symmetric undercoverage in both tails (i.e., .025) means that one-sided significance tests and confidence intervals can also be confidently used.

Hypotheses. Unless the group size was related to the group trait value, we expected the point estimate to be unbiased. We expected the one-level standard error estimates to demonstrate larger negative bias for conditions with a larger ICC or larger group size. Also, we expected the two-level standard errors to be biased for the dependent unequal group size conditions, but unbiased in other conditions. We expected the coverage to display similar trends as the bias of the standard error estimates, thus undercoverage for the one-level intervals for larger ICC conditions and over- or undercoverage for the two-level intervals for dependent unequal group sizes. The Wald-based and range-preserving confidence intervals were expected to display similar coverage values, as $H < .7$ (the value at which range-preserving methods are beneficial according to Koopman et al., in press).

Results

For all conditions, bias of the point estimate of the scalability coefficient was negligible (approximately -1%, $M = -.005$, $SD = .008$, range = $-.041; .020$). Table 1 shows the results for the two unequal group size conditions and the most similar equal group size conditions (i.e., with 20 respondents). For the dependent, unequal group size condition, the point estimate was slightly underestimated, with approximately -2% bias. .

The average bias of the one-level $SE_{\hat{H}}$ across all conditions was -.011 (approximately -31%, $SD = .014$, range $= -.073; .001$). The average bias of the two-level $SE_{\hat{H}}$ was .003 (approximately 10%, $SD = .005$, range $= -.017; .017$). There was an interaction effect of ICC and number of groups on the bias of $SE_{\hat{H}}$ for both methods, see Figure 1, left panel. The one-level $SE_{\hat{H}}$ was generally underestimated, which was more severe for fewer groups and larger ICC conditions. For (very) small ICC conditions, the one-level bias was negligible. In general, the bias of two-level $SE_{\hat{H}}$ was negligible for medium to (very) large ICC, but the

Table 1

Bias of the Point Estimate, Bias of the Standard Error, and Coverage of the 95% Confidence Interval for Conditions Equal Group Size of 20 Respondents, Independent Unequal Group Sizes, and Dependent Unequal Group Sizes for the One- and Two-Level Method.

Condition	Bias $\widehat{H}$	Bias $SE_{\widehat{H}}$		Coverage CI	
		One-level	Two-level	One-level	Two-level
Equal, 20 respondents	−.004	−.011	.004	.785	.964
Unequal, independent	−.005	−.013	.000	.753	.948
Unequal, dependent	−.011	−.011	.000	.759	.935

Note. Results were averaged across the ICC conditions and number of groups.

two-level $SE_{\widehat{H}}$ was conservative for (very) small ICC conditions. For 10 groups, the bias of $SE_{\widehat{H}}$ varied substantially for various ICC conditions. For both methods, group size had a main effect on the bias of $SE_{\widehat{H}}$ (see Figure 1, right panel). For one-level $SE_{\widehat{H}}$, negative bias increased with group size. The bias of two-level $SE_{\widehat{H}}$ was negligible for larger group sizes, but was conservative for small groups.

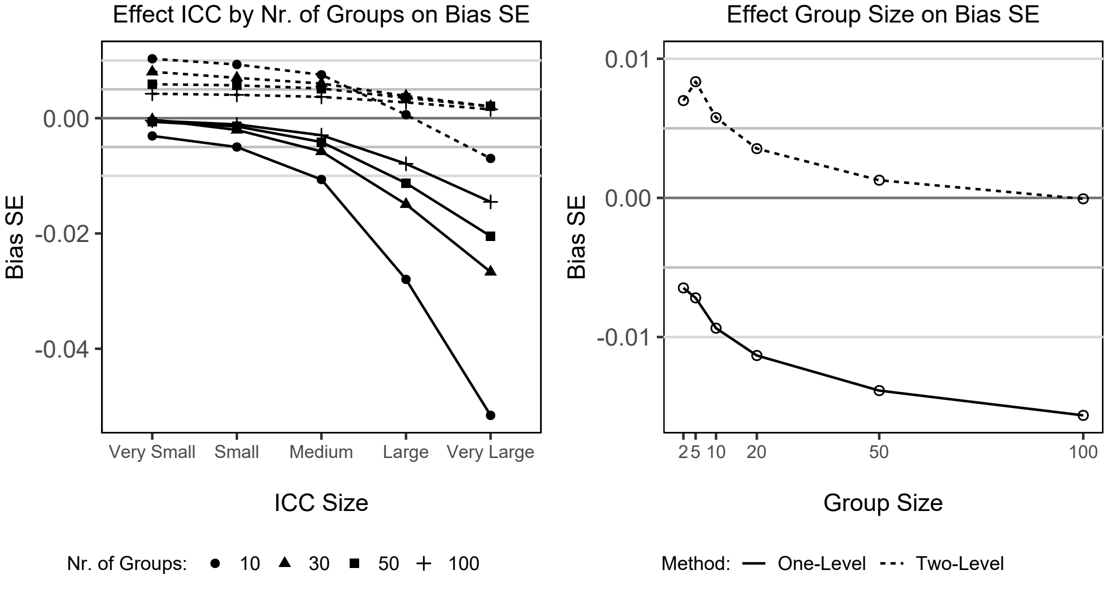

Figure 1. Left: Effect of ICC size by the number of groups on the bias of $SE_{\widehat{H}}$, for each method. Right: Effect of (equal) group size on the bias of $SE_{\widehat{H}}$. The grey lines reflect a bias of 0, (-).005, and (-).01.

Across all conditions, for the one-level Wald-based confidence interval the average coverage equalled .782 (range = .234; .963); the undercoverage was .088 in the left tail and .131 in the

right tail, respectively. For the two-level Wald-based confidence interval, the average coverage was .954 (range = .793;.995); the undercoverage was .012 and .031 in the left and right tail, respectively. The coverage of the range-preserving confidence interval was similar to the Wald-based confidence interval, although slightly less symmetrically distributed (i.e., .080 and .140 for the one-level interval and .007 and .038 for the two-level interval). Therefore, we discuss the effects of the design factors only for the Wald-based interval. There was an interaction effect of ICC and group size on the coverage of the one-level confidence interval (Figure 2, top left panel).The coverage was close to .95 for small groups and (very) small ICC

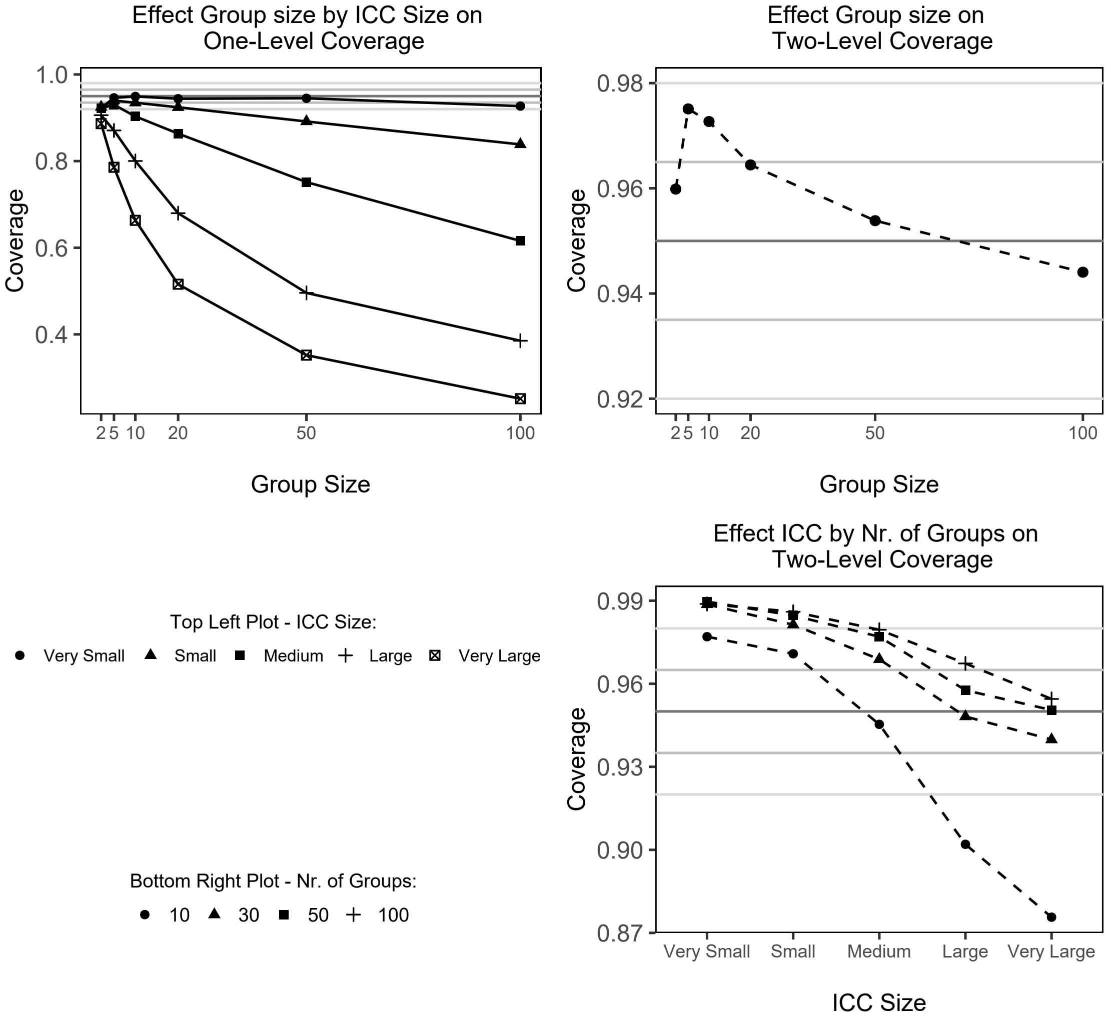

Figure 2. Top left plot: Effect of ICC size by the group size on the coverage of the one-level Wald-based confidence interval. Top right plot: Effect of group size on the two-level coverage. Bottom right plot: Effect of ICC size by the number of groups on the coverage of the two-level Wald-based confidence interval. The grey lines reflect a coverage of .92, .935, .95, .965, and .98.

conditions, but deteriorated substantially as group sizes and ICC (both) became larger. There was an interaction effect of ICC and number of groups on the two-level coverage (Figure 2, bottom right panel). The coverage improved as the ICC became larger, but deteriorated for conditions with only 10 groups. In addition, there was a main effect of group size on the two-level coverage (Figure 2, top right panel). The coverage improved for larger group sizes.

Discussion

This simulation study showed that for clustered data, the point estimates for Mokken's scalability coefficients are accurately estimated, and usually two-level standard errors and confidence intervals outperformed their one-level counterparts, especially for larger levels of within-group dependency and for larger groups. For the conditions with 10 large groups, the two-level standard error estimate was negatively biased and resulted in undercoverage of the confidence interval, for medium or larger ICCs. In such a situation, there is only little independent information present in the data, which is a possible reason for the inaccuracy of the standard error estimates (e.g., Snijders & Bosker, 2012, p. 24). Larger group sizes are recommended for better coverage rates of the two-level confidence interval. The confidence interval was somewhat asymmetric for nested data, possibly caused by skewness of the sampling distribution. As the symmetry was slightly better for the Wald-based confidence interval, we suggest using this type of interval if H is (likely) well below the upper boundary of 1. The undercoverage of the left side of the distribution was lower than the desired value of .025. This means that the one-sided significance tests that are relevant in Mokken scale analysis are likely to be somewhat conservative, with type I error rates below the nominal significance level. Our adaption of Snijders's (2001) estimation method by using averaged frequencies rather than averaged proportions to estimate the scalability coefficients and their standard errors led to accurate estimates, even when group size was related to the trait value of the group. Therefore, we recommend to use our two-level estimation method for clustered data. The two-level standard error was somewhat overestimated when the ICC is close to zero, especially for small groups. This may be explained by the underlying assumption of a multinomial distribution for each group, which cannot be accurately estimated in such situations. Alternative assumptions (e.g., a dirichlet multinomial distribution)

are computationally more complex, but may give better results.

References

Koopman, L., Zijlstra, B. J. H., De Rooij, M., & Van der Ark, L. A. (2020). Bias of two-level scalability coefficients and their standard errors. *Applied Psychological Measurement*, *44*(3), 197–214. http://doi.org/10.1177/0146621619843821

Koopman, L., Zijlstra, B. J. H., & Van der Ark, L. A. (2017). Weighted Guttman errors: Handling ties and two-level data. In L. A. Van der Ark, M. Wiberg, S. A. Culpepper, J. A. Douglas, & W.-C. Wang (Eds.), *Quantitative psychology: The 81st annual meeting of the Psychometric Society, Asheville, North Carolina, 2016.* Springer. http://doi.org/10.1007/978-3-319-56294-0_17

Koopman, L., Zijlstra, B. J. H., & Van der Ark, L. A. (2020). Standard errors of two-level scalability coefficients. *British Journal of Mathematical and Statistical Psychology*, *73*, 213–236. http://doi.org/10.1111/bmsp.12174

Koopman, L., Zijlstra, B. J. H., & Van der Ark, L. A. (in press). Range-preserving confidence intervals for scalability coefficients in Mokken scale analysis.

Kuijpers, R. E., Van der Ark, L. A., & Croon, M. A. (2013). Standard errors and confidence intervals for scalability coefficients in Mokken scale analysis using marginal models. *Sociological Methodology*, *43*(1), 42–69. http://doi.org/10.1177/0081175013481958

Kuijpers, R. E., Van der Ark, L. A., Croon, M. A., & Sijtsma, K. (2016). Bias in point estimates and standard errors of mokken's scalability coefficients. *Applied Psychological Measurement*, *40*(5), 331–345. http://doi.org/10.1177/0146621616638500

Molenaar, I. W. (1991). A weighted Loevinger H-coefficient extending mokken scaling to multicategory items. *Kwantitatieve Methoden*, *12*(37), 97-117.

Morris, T. P., White, I. R., & Crowther, M. J. (2019). Using simulation studies to evaluate statistical methods. *Statistics in Medicine*, *38*(11). http://doi.org/10.1002/sim.8086

Samejima, F. (1969). *Estimation of latent ability using a response pattern of graded scores.*

(Psychometrika monograph supplement No. 17). Psychometric Society. http://www.psychometrika.org/journal/online/MN17.pdf

Snijders, T. A. B. (2001). Two-level non-parametric scaling for dichotomous data. In A. Boomsma, M. A. J. van Duijn, & T. A. B. Snijders (Eds.), *Essays on item response theory* (pp. 319–338). Springer. http://doi.org/10.1007/978-1-4613-0169-1_17

Snijders, T. A. B., & Bosker, R. J. (2012). *Multilevel analysis: An introduction to basic and advanced multilevel modeling* (2nd ed.). Sage.

Van der Ark, L. A. (2001). Relationships and properties of polytomous item response theory models. *Applied Psychological Measurement*, *25*(3), 273–282.
